# Targeting Regulated Cell Death Pathways in COPD: Mechanisms and Therapeutic Strategies

**DOI:** 10.3390/cells14231874

**Published:** 2025-11-26

**Authors:** Hao Fu, Qian Huang, Jungang Xie

**Affiliations:** Department of Respiratory and Critical Care Medicine, National Clinical Research Center of Respiratory Disease, Key Laboratory of Pulmonary Diseases of Health Ministry, Tongji Hospital, Tongji Medical College, Huazhong University of Science and Technology, Wuhan 430030, China; fuhao200806@163.com

**Keywords:** regulated cell death, chronic obstructive pulmonary disease, apoptosis, necroptosis, ferroptosis, pyroptosis, autophagy, cuproptosis, parthanatos

## Abstract

Chronic obstructive pulmonary disease (COPD) is a progressive lung disease defined by persistent airflow limitation, chronic inflammation, and ongoing airway remodeling, and poses a substantial global health challenge. Despite its clinical significance, the underlying cellular mechanisms remain poorly defined. Regulated cell death (RCD) incorporates various kinds of cell death that are typically regulated by specific molecular pathways. Numerous new kinds of RCD have been identified outside of the traditional apoptotic pathway, like necroptosis, pyroptosis, ferroptosis, autophagy, cuproptosis, and parthanatos. To date, there is growing evidence indicating that these pathways participate in the regulation of COPD development. However, their specific roles and therapeutic relevance remain poorly understood. In this review, we discuss a summary of the molecular mechanisms by which RCD pathways influence the onset and advancement of COPD. Additionally, the therapeutic benefits of agents that target these cell death pathways in COPD treatment were described. By integrating current insights, this review aims to broaden our knowledge of the pathophysiology of COPD and offer novel approaches to treatment.

## 1. Introduction

Chronic obstructive pulmonary disease (COPD) is a prevalent and widespread respiratory condition marked by a high mortality rate, ranking as the third primary cause of death globally [1]. Parameters like smoking and exposure to toxic airborne particles, can trigger the onset of COPD [2,3]. Exposure to these risk factors induces oxidative stress [4], inflammation [5], and airway remodeling, leading to increased mucus secretion, airway obstruction, and airflow limitation [6]. These changes also disrupt alveolar structures, resulting in emphysema development and, eventually, COPD. Additionally, a crucial aspect of this process is the cell death [7,8].

**Figure 1:**
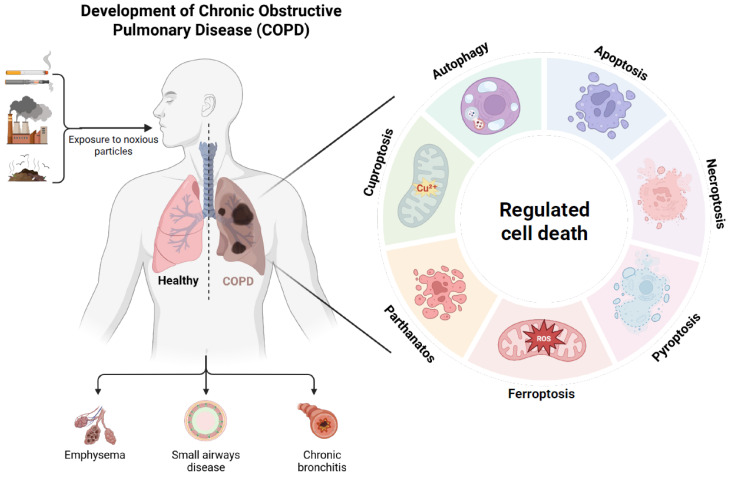
Emerging regulated cell death in COPD: apoptosis, necroptosis, ferroptosis, autophagy, pyrptosis, cuproptosis, parthanatos.

Cell death can be categorized into accidental cell death (ACD) and regulated cell death (RCD), all of which have been reported to contribute to COPD development [9] (Figure 1). Apoptosis, the classical type of RCD, significantly drives COPD progression. More recently, several non-apoptotic RCD pathways have been identified [10]. Necroptosis [11] occurs through death receptor signaling and leads to inflammatory cell death. Pyroptosis [12] is a form of inflammatory cell death triggered by inflammasome activation and gasdermin-mediated membrane pore formation. Ferroptosis [13] is an iron-dependent cell death driven by lipid peroxidation. Autophagy [14] functions as a cellular recycling mechanism, which can either promote cell survival or trigger cell death under stress. Cuproptosis [15] results from toxic copper ion accumulation, leading to cell death. Parthanatos [16] is a cell death pathway mediated by PARP1 and associated with extensive DNA damage. In COPD, the imbalance between cell death and tissue repair drives structural lung damage and emphysema, while dysregulated RCD further amplifies tissue-destructive inflammation, thereby exacerbating airway inflammation and remodeling. Given its inherent controllability, RCD represents a promising target for therapeutic intervention.

We have provided a systematic review of the cellular mechanisms and their function in COPD occurrence and progression. Furthermore, we focus on the therapeutic effects and mechanisms of pharmacological agents that target these RCD pathways, highlighting their potential applications in COPD management. The collective goal of these discoveries is to improve our knowledge of the pathophysiology of COPD and provide novel avenues for improved therapeutic development.

## 2. Apoptosis

### 2.1. Overview of Apoptosis

**Figure 2:**
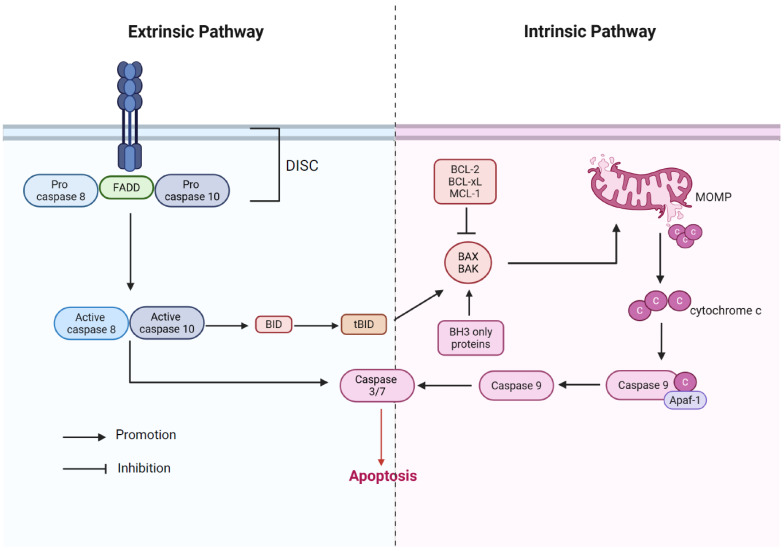
Potential molecular mechanisms of apoptosis in COPD.

A distinct form of programmed cell death, or apoptosis, as coined by Kerr, was first introduced in 1972 [17]. The hallmark morphological changes in apoptosis include cell rounding, nuclear condensation, plasma membrane blebbing, and the formation of apoptotic bodies [18]. Furthermore, it can be categorized into intrinsic apoptosis and extrinsic apoptosis [19] (Figure 2). Intrinsic apoptosis is initiated with mitochondrial outer membrane permeabilization (MOMP), which releases cytochrome c (Cyto-C) into the cytoplasm. In the cytoplasm, Cyto-C interacts with Apaf-1 to form the apoptosome, which subsequently activates caspase-9. This initiator caspase then triggers the caspases-3/7, leading to cellular disintegration [20]. Notably, this classical pathway is tightly regulated by intrinsic inhibitory factors. For example, anti-apoptotic Bcl-2 family proteins (Bcl-2, Bcl-xL, and Mcl-1) are capable of preventing MOMP by directly binding and inhibiting Bax/Bak [21], while mitochondrial fusion protein Mfn2 has been found to inhibit Bax activation through direct interaction [22]. Extrinsic apoptosis is triggered by the binding of a specific ligand to its corresponding receptor (Fas and TNF receptor superfamily member 1A (TNFRSF1A)) on the cell surface. Following ligand binding, the death-inducing signal complex (DISC) is assembled through the clustering or trimerizing of the death receptors, which recruit Fas-associated death domain (FADD) together with procaspase-8/10. To increase the apoptotic signal, the activated caspase-8/10 either directly or indirectly cleaves Bid to produce tBid, which in turn activates Bax/Bak and promotes MOMP [23]. Moreover, caspase-9 or death-associated protein kinase 1 (DAPK1) can be activated by receptor-dependent pathways involving Unc-5 Netrin Receptor B (UNC5B) and DCC Netrin 1 Receptor (DCC) after dephosphorylation induced by ligand withdrawal [18,24]. In all cases, the caspase cascade is crucial to the successful completion of apoptosis [25].

### 2.2. Apoptosis in COPD

The percentage of apoptotic cells in the lung parenchymal cells of patients with COPD is approximately 1–5%, which is more than twice the rate observed in healthy lungs [26]. Chen et al. [27] demonstrated that apoptosis is markedly increased in both bronchial and alveolar epithelial cells in emphysema mouse models generated by exposure to cigarette smoke (CS) or intraperitoneal administration of cigarette smoke extract (CSE). In addition, widespread apoptosis of pulmonary vascular endothelial cells has been noted in COPD, with evidence suggesting that this process not only represents a key pathological feature but may also actively drive disease pathogenesis [28,29]. Importantly, the ability of alveolar macrophages to phagocytize apoptotic epithelial cells is compromised in COPD, due to both intrinsic defects in macrophage function and excessive apoptotic burden. The resulting persistence of uncleared apoptotic cells results in secondary necrosis, thereby exacerbating inflammation and accelerating COPD progression [26,30]. The regulatory network governing apoptosis in COPD is complex and involves multiple intracellular signaling pathways [31]. Sonic hedgehog (Shh) signaling pathway significantly participates in the maintenance of pulmonary cellular homeostasis and suppressing apoptosis, and its dysfunction has been closely linked to COPD development [32]. Li et al. [33] reported that CSE stimulation downregulates Shh pathway-associated protein expression in MLE cells. Treatment with recombinant mouse Shh protein partially reversed apoptosis, whereas inhibition of Shh signaling with cyclosporine A (an inhibitor of the Shh pathway) enhanced apoptosis in MLE-12 cells. Furthermore, apoptosis in COPD undergoes precise regulation via multiple epigenetic mechanisms. Fu et al. [34] demonstrated that miR-613 exacerbates CSE-induced bronchial epithelial cell apoptosis and epithelial–mesenchymal transition (EMT) through the targeting of ARHGAP1, thereby contributing to inflammatory responses and airway remodeling in COPD. Similarly, Li et al. [35] showed that CSE reduces PRMT6 expression and H3R2me2a levels in pulmonary epithelial cells while upregulating Bax expression and inflammatory cytokine production. Inflammation, another central feature of COPD pathology, is also closely linked to apoptosis [10,36]. Patients with COPD present with higher levels of inflammatory mediators such as TNF-α and interferon-γ [37]. TNF-α is primarily produced by alveolar macrophages and mediates apoptosis by activating the extrinsic apoptotic pathway [38]. Interferon-γ, mainly secreted by Th1 CD4^+^ and CD8^+^ T lymphocytes, induces apoptosis in type II alveolar epithelial cells [39]. These inflammatory mediators further recruit neutrophils, lymphocytes, and macrophages to the lung parenchyma and airways. The accumulation of macrophages and neutrophils leads to protease imbalance and oxidative stress, whereas adaptive immune cells undergo proliferative expansion and promote apoptosis of alveolar epithelial cells [40].

Accumulating evidence indicates that CS can induce endoplasmic reticulum stress, resulting in the activation of the unfolded protein response (UPR), disrupts protein homeostasis, and ultimately triggers apoptosis [41]. Feng et al. [42] found that CSE promotes apoptosis in Raw264.7 macrophages by upregulating ER stress-related proteins and activating the Ca^2+^/P38/STAT1 signaling pathways, suggesting that CS-induced macrophage dysfunction may increase susceptibility to pulmonary infections in COPD. Furthermore, Chen et al. [43] demonstrated in human airway smooth muscle cells (HASMCs) that CSE upregulates ORMDL3, leading to ATF6 activation, disruption of endoplasmic reticulum calcium homeostasis, and enhanced secretion of inflammatory cytokines, thereby facilitating airway remodeling, persistent inflammation, and airflow limitation in COPD.

## 3. Necroptosis

### 3.1. Overview of Necroptosis

**Figure 3:**
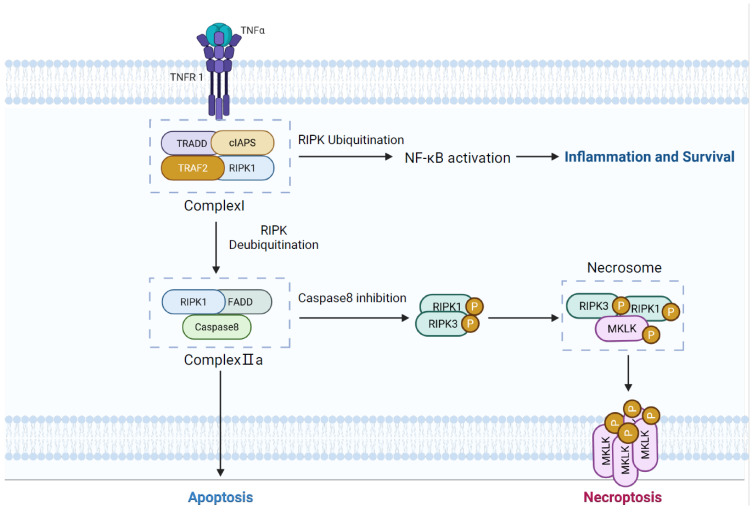
Potential molecular mechanisms of necroptosis in COPD.

Research on necroptosis commenced in 2005 [44]. Unlike apoptosis, necroptosis is caspase-independent and does not involve apoptotic body formation [45]. Instead, it is defined by the rupture of the plasma membrane, secretion of intracellular contents, and leakage of damage-associated molecular patterns (DAMPs), such as HMGB1, S100 proteins, and ATP, thereby triggering a potent inflammatory response [46]. Necroptosis is typically initiated through ligand-receptor signaling, whereby external or internal stimuli activate death receptors like TNFR1. Upon binding, TNF receptor-associated death domain protein (TRADD), TRAF2, RIPK1, and cIAPs are induced by TNFR1, thereby initiating NF-κB-mediated pro-survival signaling. Deubiquitination of RIPK1 promotes its release from complex I and subsequent formation of complex IIa with FADD and caspase-8, thereby initiating apoptosis. However, the inhibition of caspase-8 activity activates RIPK3, triggering the intracellular kinase cascade activation further [47]. Phosphorylated RIPK1 cooperates with RIPK3 and MLKL to form the necrosome, within which RIPK3 and MLKL are sequentially phosphorylated. Activated MLKL then oligomerises and inserts into the plasma membrane, generating disruptive pores that compromise membrane integrity and execute necroptosis [48,49] (Figure 3).

### 3.2. Necroptosis in COPD

Necroptosis is strongly upregulated in the lungs of patients with COPD and contributes to airway inflammation, airway remodeling, and emphysema formation [50]. Relevant studies have shown that MLKL expression in alveolar epithelial cells and macrophages of patients with severe disease is elevated, accompanied by increased levels of phosphorylated RIPK3(p-RIPK3) and phosphorylated MLKL(p-MLKL) in lung tissue [50]. Conversely, the absence of RIPK3 or MLKL reduces CS-induced macrophage death, thereby alleviating airway inflammation, remodeling, and the pathological progression of emphysema [51]. Furthermore, RIPK1 deficiency diminishes smoking-induced pulmonary cell apoptosis and necrotic cell death, thereby alleviating airway inflammation, remodeling, and emphysema [52] while also preventing cigarette-induced impairment of mucociliary clearance [53]. The specific mechanisms by which necroptosis contributes to COPD pathogenesis may include aberrant upregulation of pro-inflammatory mediators, inefficient removal of dead cells, and promoting oxidative stress. For example, Zeng et al. [54] demonstrated that miR-21 knockout reduces necrotic apoptosis-associated proteins (TNFR1 and p-MLKL) in bronchial epithelial cells, concurrently lowering inflammatory cytokine levels and thereby ameliorating COPD symptoms. According to Wang and colleagues [55], GRP78 facilitates CSE-induced inflammation of airway epithelial cells and excessive mucus production by upregulating necrotic apoptosis and subsequently activating NF-κB and AP-1 pathways. In addition, Chen and associates [56] showed that impaired macrophage clearance of necrotic cells and DAMPs reduces phagocytic efficiency, thereby amplifying pro-inflammatory factor expression and ultimately triggering pathological inflammatory responses. Another study further reported that CSE induces necroptosis in bronchial epithelial cells, triggering the secretion of mitochondrial DNA (mtDNA) into the cytoplasm and extracellular space, which in turn activates inflammatory signaling [57].

## 4. Ferroptosis

### 4.1. Overview of Ferroptosis

**Figure 4:**
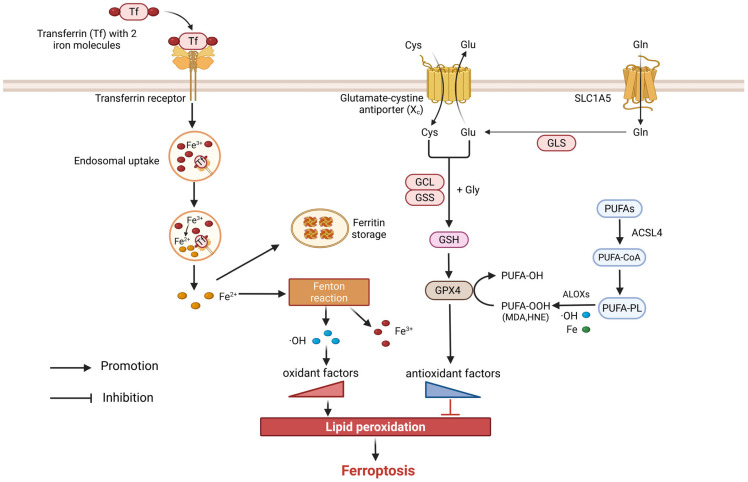
Potential molecular mechanisms of ferroptosis in COPD.

Ferroptosis is a controlled type of RCD that is initiated by oxidative disturbances within the intracellular microenvironment and defined by its reliance on iron and lipid peroxidation. Morphologically, it is characterized by mitochondrial shrinkage, increased membrane density, decreased cristae density, and eventual rupture of the mitochondrial membrane [58]. Its mechanisms are complex and involve interactions among iron metabolism, glutathione metabolism, and lipid peroxidation (Figure 4). In iron metabolism, Ferric iron (Fe^3+^) is taken up by cells via transferrin receptors (TFRs), where it is converted to ferrous iron (Fe^2+^) within endosomes, and subsequently transported into the labile intracellular iron pool. Under pathological conditions such as infection or inflammation, disruption in iron homeostasis may result in an accumulation of iron within cells. Excess iron accelerates lipid peroxidation of polyunsaturated fatty acids (PUFAs), ultimately driving ferroptosis. The cystine/glutamate antiporter system Xc^−^ is responsible for maintaining redox balance. This system consists of the light chain subunit xCT (SLC7A11) and the heavy chain subunit CD98hc (SLC3A2), which facilitates the import of cystine in exchange for glutamate [59]. Intracellular cystine is enzymatically converted into glutathione (GSH), which, as an electron donor, reduces phospholipid hydroperoxides (PL-OOH) to their corresponding alcohols through the action of glutathione peroxidase 4 (GPX4), thereby mitigating lipid toxicity and inhibiting ferroptosis [60]. Ferroptosis is a process that takes place via an iron-catalyzed lipid peroxidation mechanism. This process can be triggered by both non-enzymatic (Fenton reaction) and enzymatic mechanisms (lipoxygenases, ALOXs). The Fenton reaction, mediated by Fe^2+^, produces reactive oxygen species (ROS) such as hydroxyl radicals. These ROS engage in lipid peroxidation reactions with phospholipid-containing PUFAs to generate lipid peroxidation products (PL-PUFA-OOH) such as malondialdehyde (MDA) and 4-hydroxynonenal (HNE), which disrupt cell membrane fluidity and permeability, leading to cell death. In parallel, ALOXs, a family of non-heme iron-containing enzymes, catalyze the PUFA oxygenation to produce PL-PUFA-OOH, further amplifying lipid peroxidation and promoting ferroptosis.

### 4.2. Ferroptosis in COPD

In 2012, B. Stockwell introduced the concept of ferroptosis [61] and was implicated in the regulation of COPD formation in 2019 [62]. Growing evidence reveals that patients with COPD display noticeably raised levels of ACSL4, LPCAT3, and lipid peroxidation products compared to healthy controls [63]. CS increases the expression of the key ferroptosis protein NCOA4 in COPD, promoting epithelial ferroptosis, iron release, and iron accumulation. Excess intracellular iron enhances ROS production through the Fenton reaction, further depleting GPX4, exacerbating lipid peroxidation, and inducing cell death, airway inflammation, airway remodeling, and emphysema development [64]. Ferroptosis is therefore characterized by iron overload, excessive lipid peroxidation, and disruption of the system Xc^−^/GSH/GPX4 antioxidant axis [65], collectively driving cellular damage and contributing to COPD pathogenesis.

As previously mentioned, ACSL4 is pivotal in lipid peroxidation and acts as a crucial regulator of ferroptosis. Wang et al. [66] reported that both Lv-shACSL4-treated COPD mouse models and siACSL4-transfected cells effectively suppressed ACSL4 expression, decreased lipid peroxidation, and downregulated ferroptosis markers in vivo and in vitro. This suggests that ACSL4 is a key factor in ferroptosis in COPD. Liu and colleagues [67] found that the deubiquitinating enzyme USP8 was downregulated in COPD models. Its overexpression activated the OTUB1/SLC7A11 pathway, enhancing cystine uptake and GSH synthesis to maintain GPx4 activity. This, in turn, suppressed ACSL4 expression, alleviating lung inflammation and airway remodeling. Epigenetic regulation influences the pathogenesis of COPD by affecting ferroptosis, offering new therapeutic directions. TET2, a DNA demethylase, catalyzes cytosine hydroxymethylation and chromatin remodeling to regulate gene expression, playing a crucial epigenetic role in myeloid hematopoiesis, inflammatory responses, and various pulmonary diseases. Zeng [68] et al. found that TET2 downregulation induces hypermethylation of the GPx4 promoter, impairing antioxidant defenses and exacerbating ferroptosis. Notably, co-treatment with the demethylating agent 5-AZA and the antioxidant NAC synergistically reduced ferroptosis and lung injury, suggesting that combining methylation inhibitors with antioxidants could provide a potentially new therapeutic approach for COPD. Xia et al. [64] identified the m6A-modified circSAV1/YTHDF1/IREB2 axis as the key driver of CS-induced ferroptosis in lung epithelial cells. This pathway promotes IREB2 mRNA translation, disrupts iron homeostasis, and exacerbates lipid peroxidation. Targeting circSAV1 or inhibiting key molecules in this axis significantly mitigated alveolar enlargement and airway remodeling in COPD mouse models.

## 5. Autophagy 

### 5.1. Overview of Autophagy

**Figure 5:**
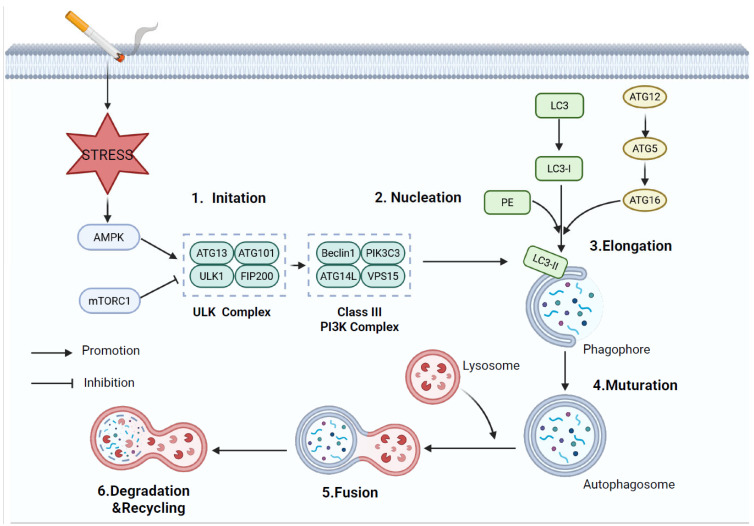
Potential molecular mechanisms of autophagy in COPD.

Christian de Duve termed his observation of the lysosome-dependent degradation in cells, autophagy, in 1963 [69]. Autophagy is a highly conserved, pivotal biological process in eukaryotic cells that involves the breakdown of and recycling of damaged organelles, misfolded proteins, or invading pathogens. This process occurs within a double-membraned autophagosome, which fuses with lysosomes for degradation [70]. Autophagy-dependent cell death (ACD) represents a type of RCD governed by autophagic molecular processes. The Nomenclature Committee on Cell Death (NCCD) states that ACD is fundamentally different from classical types of cell death, like apoptosis and necrosis, and requires activation of the autophagy pathway or its key molecular components [19,71]. Based on substrate transport mechanisms, autophagy is primarily categorized into three major types: macroautophagy, microautophagy, and chaperone-mediated autophagy. Of these, macroautophagy represents the predominant form, commonly referred to simply as autophagy [72]. This process is strictly governed by a set of autophagy-related (ATG) proteins and occurs through five stages: induction, nucleation, elongation, maturation, and degradation (Figure 5). Under stress conditions, such as smoking, activation of AMPK or inhibition of mTORC1 leads to dissociation of mTORC1 from UNC-51-like kinase 1 (ULK1), resulting in ULK1 dephosphorylation and activation [73]. The activated ULK1 complex (ULK1-ATG13-FIP200-ATG101) subsequently recruits and activates the downstream Class-III PI3K complex (Beclin1-ATG14-VPS15-VPS34), which generates phosphatidylinositol 3-phosphate (PI3P) to initiate autophagosome nucleation [74]. Subsequently, microtubule-associated protein 1 light chain 3 (LC3) is cleaved by the ATG4 protease into LC3-I, which is then conjugated to phosphatidylethanolamine (PE) via the ATG12-ATG5-ATG16L1 ubiquitin-like conjugation system, forming membrane-bound LC3-II. LC3-II facilitates the extension and invagination of the phagophore membrane, ultimately enveloping substrates for degradation to form mature autophagosomes [75]. Autophagosomes are then fused with lysosomes to generate autolysosomes, which are responsible for the lysis of autophagosome contents using acidic hydrolases. The resulting metabolites, including amino acids and fatty acids, are recycled into cellular metabolic pathways, supplying both energy and biosynthetic precursors [76].

### 5.2. Autophagy in COPD

Accumulating evidence indicates that autophagy is dysregulated in COPD patients, exhibiting extremely dynamic and context-dependent bidirectional regulation. Compared to age-matched non-smokers, peripheral lung tissue from patients with severe COPD exhibits increased levels of p62, LC3, and protein aggregates, suggesting impaired autophagy in COPD [77]. Consistently, p62 accumulation in peripheral lung tissue positively correlates with pulmonary function severity and is closely associated with upregulation of LC3 and BICD1 expression [78]. In addition, alveolar macrophages from COPD patients and smokers display increased autophagosome formation, further supporting the notion that defective autophagic flux contributes to COPD pathogenesis [79]. Galectin-8 functions as a danger receptor, recognizing destroyed intracellular host vesicles that are damaged and recruits the autophagy adaptor protein NDP52 to facilitate forming and breaking down specific autophagosomes. Yuta Kono and associates [80] found lung homogenates from individuals with COPD demonstrated elevated galectin-8 and NDP52 levels, with significantly increased serum galectin-8 levels in those experiencing frequent acute exacerbations. At the cellular level, it has been demonstrated that CSE disrupts autophagic flux in U937 macrophage-like cells, resulting in the intracellular concentration of NDP52 and galactosidase-8. Conversely, chronic smoke exposure may also induce excessive autophagy activation, resulting in alveolar structural disruption, ciliary shortening, and impaired mucus clearance, thereby exacerbating airway remodeling and emphysema formation [81]. Bulk and single-cell RNA sequencing datasets from COPD and non-COPD tissues were examined by Liao et al. [82], who found significant upregulation of autophagy-related genes in COPD. Moreover, autophagy in macrophages and monocytes displayed extensive interactions with other cell types, highlighting its potential role as a key regulatory process in intercellular communication. Chen et al. [83] further demonstrated that mTOR deficiency enhances inflammatory responses, whereas autophagy inhibition via Atg5 deletion attenuates inflammation, suggesting that autophagy activation contributes to particulate aerosol-induced pulmonary inflammation.

Autophagy can be classified as either selective or non-selective forms based on the specificity of the degraded material [84]. Mitophagy, a selective type, specifically removes dysfunctional mitochondria to prevent ROS accumulation and apoptosis [85]. Abnormal activation of mitophagy is considered a key mechanism by which CS promotes COPD development. Yang et al. [86] demonstrated that ARSK alleviates COPD by inhibiting autophagy and restricting excessive mitophagy through suppression of Parkin Ser65 phosphorylation. Correspondingly, Wei and colleagues [87] revealed in a CS-induced mouse model that STAT3 activation enhances PINK1/Parkin pathway activity by upregulating TGF-β1, with excessive mitochondrial autophagy further inducing epithelial–mesenchymal transition (EMT) and small airway wall thickening. Beyond CS, environmental pollutants PM2.5 and microplastics also modulate mitochondrial autophagy through distinct mechanisms. Wang et al. [88] revealed that PM2.5 uncouples Klotho-mediated inhibition of IGF-1R, leading to Parkin-dependent mitophagy defects. Impaired clearance of damaged mitochondria subsequently triggers oxidative stress. Conversely, Wei et al. [89] demonstrated that polystyrene microplastics (PS-MPs) activate autophagy-dependent ferroptosis (ADF) via mitochondrial ROS accumulation, where NCOA4-mediated ferritinophagy releases free iron, exacerbating lipid peroxidation. These outcomes emphasize the complex and context-dependent function of mitochondrial autophagy in COPD. Insufficient mitophagy promotes oxidative stress, whereas excessive activation disrupts mitochondrial homeostasis. Despite operating through distinct mechanisms, both processes converge to drive airway remodeling and COPD progression [86].

## 6. Pyroptosis

### 6.1. Overview of Pyroptosis

**Figure 6:**
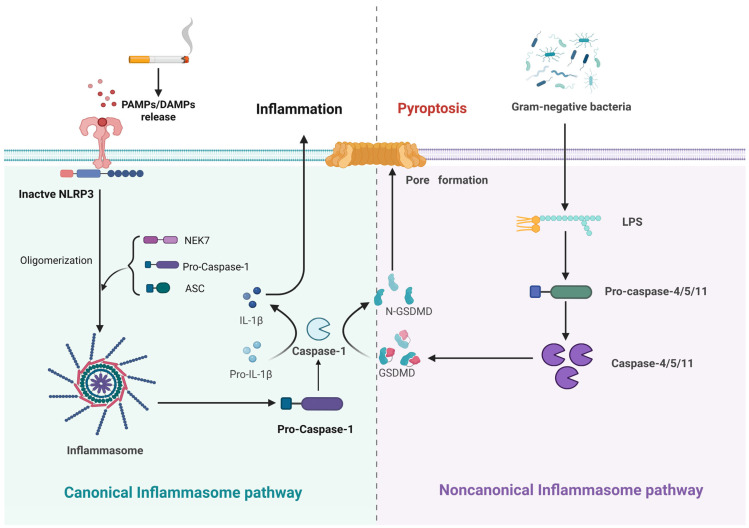
Potential molecular mechanisms of pyrptosis in COPD.

The term “pyroptosis” was first introduced in 2005 [63]. Pyroptosis represents a type of RCD modulated by gasdermin (GSDM) proteins (Figure 6). It is structurally defined by chromatin condensation, cytoplasmic vesicle formation, and the rupturing of the plasma membrane. External factors like smoking and PM2.5 can trigger the release of pathogen-associated molecular patterns (PAMPs) or DAMPs. These molecules are recognized by pattern recognition receptors (PRRs), which trigger and induce the formation of inflammasomes [90,91,92,93,94]. Two major classes of PRRs involved in pyroptosis in COPD are nucleotide-binding domain leucine-rich repeat-containing proteins (NLRs) and AIM2-like receptors [95,96]. The formed inflammasome cleaves pro-caspase-1 into an active form, which then processes GSDM into the N-terminal fragment (GSDM-N) and matures pro-IL-1β and pro-IL-18. GSDM-N produces pores in the plasma membrane, enabling IL-1β/IL-18 release and amplifying inflammation [97,98,99]. In addition to this canonical pathway, pyroptosis can also occur through inflammasome-independent mechanisms. Intracellular lipopolysaccharide (LPS) activates caspase-4/5/11 directly, which then induces GSDMD cleavage and the release of GSDMD-N-terminal fragments [4,5].

### 6.2. Pyroptosis in COPD

Numerous chronic respiratory conditions, including COPD, have been linked to pyroptosis [100,101]. By 2013, studies demonstrated that IL-18 expression induces pulmonary inflammation, emphysematous changes, excessive mucus secretion, and fibrotic alterations in the blood vessels and airways of mice [102]. Nevertheless, the exact function of pyroptosis in COPD is still not fully determined. Faner and associates observed markedly elevated NLRP3, IL-1β, and IL-18 expressions in lung tissue from patients with stable COPD [103]. Subsequent studies further revealed that multiple pyroptosis-associated molecules, including NLRP3, caspase-1, and IL-18, are further upregulated during acute exacerbations of COPD [96,97]. Nicotine, a major constituent of CS, has been identified as a major driver of pyroptotic pathways [104]. Wang et al. [90] reported that nicotine exposure upregulates pyroptosis-associated molecules in 16HBE cells, potentially contributing to COPD progression. Similarly, Zhang et al. [105] demonstrated that CSE induces pyroptosis in 16HBE cells via the classical ROS/NLRP3/caspase-1 pathway, leading to GSDMD cleavage and release of IL-1β and IL-18. Rao et al. [106] further found that TRPV4 upregulation promotes pyroptosis by activating caspase-1 and increasing GSDM-N protein expression. Mechanistically, TRPV4 modulates the Ca^2+^/NLRP3/caspase-1 axis, while its inhibition attenuates NLRP3 inflammasome activation, lactate dehydrogenase release, and inflammatory cytokine production. While ferroptosis is defined by lipid peroxidation, lipid peroxidation can also initiate pyroptosis. Hou et al. [107] demonstrated that inhibiting xCT ubiquitination alleviates lipid peroxidation-mediated pyroptosis in COPD patients through downregulating the xCT/GSH/GPx4 axis. In vitro, Mo et al. [108] established a COPD cell model by treating the human lung fibroblast line MRC-5 with lipopolysaccharide and demonstrated that lncRNA GAS5 overexpression facilitates pyroptosis in pulmonary fibroblasts through activation of the miR-223-3p/NLRP3 axis. However, other studies yielded conflicting findings. One study reported that CSE suppresses NLRP3 inflammasome expression and its downstream cytokines IL-1β and IL-18 at the transcriptional level, while simultaneously activating caspase-1 via the noncanonical TLR4/TRIF/caspase-8 pathway [109]. This unique caspase-1 activation reduced glycolytic flux in macrophages, suggesting distinct and context-dependent cellular responses to CSE that require further investigation.

## 7. Other Forms of RCD

### 7.1. Cuproptosis

Cuproptosis (copper-induced cell death) denotes a recently identified form of programmed cell death, first described in 2022 by Tsvetkov and colleagues [110]. Its core mechanism involves the excessive binding of intracellular copper ions (Cu^2+^) to lipoylated proteins in the mitochondrial tricarboxylic acid (TCA) cycle, forming protein aggregation and destabilizing iron-sulfur (Fe-S) cluster proteins. This cascade induces proteotoxic stress, impairs mitochondrial respiration, and ultimately triggers cell death [111]. In contrast to other types of cell death, like ferroptosis and apoptosis, cuproptosis depends on copper–protein interactions and TCA cycle dysfunction, and cannot be blocked by conventional cell death inhibitors [112]. Evidence from COPD patients and cigarette smoke exposure models indicates significant dysregulation of cuproptosis-associated genes, including GLS, DLD, CDKN2A, and DLST. Among these, GLS expression is reduced and correlates with lung function decline, whereas DLD and CDKN2A are upregulated in response to smoking and related factors [113,114]. These genes not only serve as diagnostic biomarkers for COPD but are also implicated in copper homeostasis and cuproptosis-associated pathways, thereby offering potential avenues for targeted therapy [115]. Nevertheless, the exact mechanisms by which cuproptosis participates in the development of COPD pathogenesis remain unresolved, underscoring the need for further mechanistic studies and the development of targeted intervention strategies.

### 7.2. Parthanatos

Parthanatos is classified as a type of RCD that is reliant on Poly(ADP-ribose) (PAR) polymerase-1 (PARP-1). PARP1 is a chromatin-associated nuclear protein that significantly participates in DNA repair during strand breaks [116,117,118]. Parthanatos can be induced by oxidative stress, such as that caused by smoking [119]. ROS can induce DNA damage, leading to the overactivation of PARP1 and the accumulation of PAR. This cascade promotes the nuclear translocation of mitochondrial proteins, including apoptosis-inducing factor (AIF) and Endonuclease G (EndoG). Once in the nucleus, AIF and EndoG cause chromatin dissolution and cell death [120]. Studies have shown that the EndoG inhibitor BMN673 can reduce levels of AIF and EndoG in epithelial cells [121]. However, BMN673’s potential role in modulating parthanatos and attenuating cell death in COPD remains a subject for further investigation [121].

## 8. Therapeutic Potential

### 8.1. Targeting Apoptosis

Research has demonstrated that multiple compounds confer anti-apoptotic effects by selectively modulating key pathways involved in apoptosis (Table 1). As previously noted, Bax and Bcl-2 are critical proteins of the Bcl-2 protein family, functioning as pro-apoptotic and anti-apoptotic factors, respectively, and jointly mediating the process of programmed cell death through the mitochondrial apoptosis pathway. Asiaticoside activates the PGC-1α/Nrf2 pathway, resulting in downregulation of Bax and upregulation of Bcl-2. This action not only mitigates inflammation but also suppresses EMT, thereby reducing small airway wall thickening in CS-induced lung tissue [122]. Another study showed that Korean Red Ginseng can also counteract the toxicity of cigarette smoke condensate (CSC), significantly reducing apoptosis levels in NCI-H292 cells and mouse lung tissue by inhibiting the overactivation of the VEGF/PI3K/AKT pathway, which alleviates alveolar damage and emphysema [123]. Caspase-3, the central executioner of apoptosis, integrates signals from multiple pathways and induces DNA fragmentation, leading to cell death. Formononetin (FMN) [124] and N-acetyl-L-cysteine (NAC) [125] were reported to inhibit the AhR/CYP1A1/AKT/mTOR pathway, while NAC also replenishes GSH and suppresses the p53 pathway. Both compounds significantly reduce Caspase-3 activation, alleviating alveolar damage and demonstrating therapeutic potential for restoring lung function.

Mounting evidence further highlights oxidative stress as a key regulator of apoptosis, making it an attractive therapeutic target [129]. Anthrahydroquinone-2,6-disulfonate (AH_2_QDS) is a labdane diterpene lactone compound derived from Andrographis paniculata that exhibits anti-inflammatory, antioxidant, and antiviral activities. Li and colleagues [126] demonstrated that AH_2_QDS activates the Keap1/Nrf2 pathway, upregulating downstream antioxidant proteins HO-1 and NQO1, which enhances cellular antioxidant capacity, reduces inflammation, and decreases apoptosis in rat lung tissue induced by LPS and CS, ultimately contributing to the restoration of lung function. Progesterone (P4), a key steroid hormone, was documented to also protect breast cancer cells and uterine leiomyoma tissue from oxidative stress-induced apoptosis [130]. Li et al. [128] further reported that P4 activates the c-MYC/SIRT1/PGC-1α pathway to maintain the function of the mitochondria and reduce H_2_O_2_-induced apoptosis in BEAS-2B cells and airway smooth muscle cells. This suggests that P4 may have therapeutic potential beyond reproductive health, particularly in ameliorating the symptoms and advancement of COPD. In addition, Gaylussacin, a potential therapeutic agent for COPD, markedly suppresses MMP-12 expression and activity in macrophages by activating the SIRT1 pathway, thereby mitigating pulmonary inflammation, oxidative stress, and apoptosis in models induced by lead/cadmium (Pb/Cd) or porcine pancreatic elastase (PPE). These effects lead to improved lung function and slowed disease progression. Additionally, its favorable oral bioavailability and safety profile further support its therapeutic promise [127].

### 8.2. Targeting Necroptosis

Intervening in necroptosis-related key kinases has shown therapeutic potential in COPD (Table 2). GSK’547 is a RIPK1 inhibitor that significantly reduces elastase-induced emphysema and lung function decline, diminishes CS-induced peri-bronchial inflammatory cell infiltration, airway collagen deposition, and emphysema-like changes [52]. Necrostatin-1 (NEC-1), another RIPK1 inhibitor, has been reported to suppress cigarette smoke-induced inflammatory cytokine secretion from bone marrow–derived macrophages (BMDMs) [51]. However, Chen et al. [56] suggested that NEC-1 does not alleviate cigarette-induced emphysema and airway inflammation, and hypothesized that in COPD, there may exist mechanisms of RIPK3 activation that are independent of RIPK1. In contrast, RIPK3 inhibitor GSK’872 significantly reduces smoking-induced lung macrophage and epithelial cell death [51,56] and markedly improves COPD-related pathological changes [56]. The discrepancy in findings regarding NEC-1’s effects on CSE-induced cell death may result from differing assessment methods. To further clarify RIPK1’s role, gene knockout models are needed. Notably, Hannelore et al. [52] further employed single-cell sequencing and demonstrated elevated RIPK1 expression in multiple lung cell types in both COPD patients and experimental mouse models. Genetic or pharmacological inhibition of RIPK1 using GSK’ 547 markedly lowered airway inflammation following acute and subacute exposure to CS, reduced apoptosis and necrosis induced by chronic exposure, and mitigated airway remodeling and emphysema. Collectively, these findings reveal that RIPK1 is upregulated in COPD and represents a potential therapeutic target, though its interaction with RIPK3 remains to be fully defined.

Additionally, traditional Chinese medicine extracts targeting necroptosis have also shown promising effects in COPD treatment. Theaflavin 3,3′ digallate (TF-3), an extract from black tea, significantly improves CS-induced lung inflammation and emphysema. TF-3 inhibits necroptosis by reducing RIPK3 and MLKL phosphorylation [131]. Thymoquinone (Tq), an active ingredient extracted from black cumin, inhibits RIPK1 and RIPK3 expression, reduces MLKL phosphorylation, and attenuates CSE-induced epithelial cell death. Its protective effects on epithelial cells warrant further investigation in preclinical studies [132].

### 8.3. Targeting Ferroptosis

Various chemical agents targeting ferroptosis reduce cell death and ameliorate COPD pathology [133,134,135] (Table 3). Ferrostatin-1 (Fer-1) is a ferroptosis inhibitor. Deferoxamine (DFO) chelates iron, reducing intracellular iron levels. Both Fer-1 and DFO markedly reduce CS-induced epithelial cell death, which in turn decreases inflammation and potentially helps mitigate inflammatory cell infiltration and lung injury in COPD [135,136,137,138]. In addition, Fer-1 inhibits macrophage M2 polarization induced by epithelial cells [139]. Plant extracts can also improve COPD pathology by regulating ferroptosis. Ginsenoside Rg1 [140], Ginkgo biloba extract (GBE) [141], and Sea buckthorn extract (SBE) [142] can effectively reduce inflammatory cell infiltration and promote alveolar repair by triggering the Nrf2/SLC7A11/GPX4 pathway and inhibiting the occurrence of ferroptosis. Acacetin [143], Curcumin (CUR) [136], scutellarein (STR) [36], and dihydroquercetin (DHQ) [144] are natural compounds that significantly inhibit CS-induced ferroptosis, thereby alleviating airway inflammation and ameliorating emphysema. CUR and DHQ further enhance cellular antioxidant capacity by promoting System Xc^−^ and glutathione (GSH) expression [36,136]. In addition to promoting GSH and NRF2, DHQ also inhibits ALOX15, reducing lipid peroxidation [144]. Other antioxidants, including hydrogen sulfide (H_2_S) and sodium pyruvate (NaPyr), exert similar protective effects by upregulating NRF2, GSH, and GPX4, suppressing CS-induced epithelial ferroptosis and inflammation, and alleviating PM2.5-triggered airway injury [145]. Moreover, Lipoxin A4 (LXA4), an endogenous lipid mediator produced through lipoxygenase-dependent metabolism of arachidonic acid, exhibits both anti-inflammatory and antioxidant effects [146]. Li et al. [147] demonstrated that LXA4 activates the ALX/FPR2 receptor and inhibits p38/MAPK phosphorylation, suppressing ferroptosis and inflammatory responses while improving lung function and tissue integrity. However, clinical translation of LXA4 is hindered by its rapid inactivation and short half-life. Consequently, developing stabilized LXA4 analogs represents a potentially viable treatment approach for COPD.

### 8.4. Targeting Autophagy

Various agents can precisely modulate the balance between LC3B II and p62 expression, restoring autophagic homeostasis and alleviating lung injury (Table 4). The traditional Chinese medicine formula Bufei Yishen Formula (BYF) and its active components effectively upregulate LC3B II and downregulate p62, activating the autophagy pathway to promote the clearance of damaged organelles and suppress cellular senescence. It also mitigates inflammation and oxidative stress, preserves epithelial barrier function, and improves lung performance [148,149]. Similarly, natural compounds such as dihydromyricetin (DHM) [150] and quercetin [151], as well as the sildenafil analog vardenafil [152], can restore autophagy function, clear CS-induced lung tissue damage components, and improve lung function. DHM additionally reduces mucus hypersecretion and repairs ciliary dysfunction by rebalancing autophagy [150]. Conversely, when excessive autophagy contributes to cell injury, diindolylmethane (DIM) has demonstrated regulatory effects. Jung et al. [153] showed that in HEL299 human lung fibroblasts exposed to cadmium, a recognized risk factor for COPD, DIM suppresses excessive autophagy and attenuates autophagy-related cell death, revealing its potential as a viable treatment candidate for cadmium-associated diseases, including COPD.

Pharmacological regulation of mitophagy has also been recognized as a therapeutic avenue for COPD, either by repressing its overactivation or restoring its impaired activity. Puerarin, an isoflavone derived from kudzu root, exhibits antioxidant and anti-apoptotic properties. By activating the PI3K/AKT/mTOR pathway, it inhibits CSE-induced excessive mitophagy, as demonstrated by Wang et al. [154], thereby rescuing mitochondrial function and protecting bronchial epithelial cells from CSE-induced injury, providing a novel potential strategy for COPD therapy. In contrast, hydrogen sulfide (H_2_S) has been reported to enhance impaired Parkin-mediated mitophagy in a PM2.5 model by upregulating Klotho expression and inhibiting IGF-1R, clearing abnormal mitochondria, and alleviating cellular senescence and inflammation [88]. These effects significantly mitigate emphysema and airway inflammation, inhibit COPD progression, and underscore the potential clinical applications of H_2_S-based therapies. Collectively, these findings emphasize the importance of maintaining mitophagy at an appropriate level to preserve mitochondrial homeostasis and slow disease progression.

### 8.5. Targeting Pyroptosis

The strategy of suppressing pyroptosis by inhibiting NLRP3 inflammasome activation, blocking inflammatory caspase maturation, and preventing GSDMD cleavage offers a promising therapeutic approach for COPD (Table 5). A variety of natural plant extracts, including Resveratrol [155], DHM [156], Schisandrin A (SchA) [157], Tanshinone(TS) [158], grape seed proanthocyanidin extract(GSPE) [159], Daphnetin(Daph) [160], osthole [161], the flower buds of *Tussilago farfara* L (FTF) [162], (−)-Epicatechin(EC) [163], and isoforskolin (ISOF) [164], have demonstrated therapeutic efficacy in COPD treatment. These extracts inhibit lung cell pyroptosis by suppressing the NLRP3 inflammasome, alleviating airway inflammation and emphysema, and improving lung function. For instance, DHM can directly bind to and stabilize SLC7A11, enhancing glutathione synthesis and subsequently inhibiting lipid peroxidation and pyroptosis [156]. SchA also activates the NRF2/HO-1 signaling pathway, an important target in COPD treatment [157], whereas TS inhibits both NF-κB activation and the secretion of inflammatory cytokines IL-6 and IL-8 [158]. Daph additionally reduces airway inflammation and mucus hypersecretion resulting from PM2.5 exposure or cigarette smoke [160]. Consistent with these findings, traditional Chinese medicine exhibits complex pharmacological properties, particularly in regulating pyroptosis. Both Astragaloside IV [165] and Tianlong Kechuanling (TL) [166] were reported to control the NLRP3-GSDMD signaling pathway, thus participating in the regulation of pyroptosis. Notably, TL inhibits pyroptosis in pulmonary vascular endothelial cells, alleviates inflammatory responses and vascular remodeling, and consequently reduces pulmonary arterial hypertension while improving pulmonary function. It shows great clinical promise for COPD complicated by pulmonary arterial hypertension.

In addition, other chemical agents targeting pyroptosis have also shown potential in COPD therapy [168]. Hydrogen sulfide, a chemical agent, alleviates smoking-induced lung inflammation [169,171]. Magnesium isoglycyrrhizinate (MgIG), a derivative of glycyrrhizic acid, mitigates smoking and LPS-induced lung function decline, suppressing airway inflammation, remodeling, and emphysema [172]. A range of specific inhibitors has currently been developed primarily for use in experimental research settings, such as the NLRP3 inhibitor MCC950 [170] and the caspase-1 inhibitor VX-765 [105], both of which markedly attenuate lung inflammation. As previously noted, GSDMD is the central executor of pyroptosis, mediating lytic and inflammatory cell death. Gao et al. [167] demonstrated that disulfiram (DSF) directly targets GSDMD and, through covalent binding, prohibits the pore-forming activity of its N-terminal fragment (GSDMD-NT), thus preserving airway epithelial barrier integrity and attenuating ozone-induced COPD pathology and lung function impairment. Given its established safety profile as an approved drug, DSF holds significant translational potential as a therapeutic agent for COPD. However, its efficacy and mechanism of action in CS-induced models should be well-established in future research to support clinical application. Notably, although AIM2-mediated pyroptosis also participates in the development of COPD [96,173], little is currently known about pharmacological agents capable of regulating the AIM2 inflammasome.

## 9. The Future of Translational Research in RCD for COPD

Beyond their mechanistic roles, RCD pathways offer significant translational potential. Insights from therapeutic targeting of RCD in oncology provide a conceptual framework for developing novel COPD treatments, particularly through the identification of prognostic biomarkers and the design of pharmacological modulators. In colorectal and hepatocellular carcinoma [174,175], RCD-based prognostic models not only facilitate patient stratification but also uncover differential sensitivities to chemotherapy and immunotherapy. Comparable strategies are now being applied to respiratory diseases. For instance, a seven-gene “cell death score” stratifies risk and predicts therapy response in idiopathic pulmonary fibrosis [176], while a pyroptosis-related gene set was shown to predict 28-day survival and correlates with immune cell infiltration in sepsis-induced acute respiratory distress syndrome (ARDS) [177]. In COPD, serum ferroptosis-related biomarkers such as sTfR1 and GPX4 have been identified as independent predictors of exercise capacity and exacerbation risk [178]. These findings highlight the potential of molecular signatures related to regulated cell death to serve as tools for risk stratification, disease monitoring, and personalized therapy in COPD.

Encouragingly, both preclinical and early clinical studies have begun to validate several regulators of RCD as potential therapeutic targets. NLRP3 inhibitors, such as NT-0796 [179] and DFV890 [180], have demonstrated favorable safety and pharmacodynamic profiles, including central nervous system penetration and dose-dependent suppression of IL-1β in early human trials. In parallel, activators of the Nrf2 pathway such as dimethyl fumarate (DMF) and its metabolite monomethyl fumarate (MMF) exhibit combined antioxidant and anti-inflammatory properties [181]. In experimental models of COPD, DMF attenuates oxidative stress, reduces macrophage infiltration, and partially reverses emphysematous changes. Given its established clinical use in multiple sclerosis [182] and psoriasis [183], DMF represents a particularly promising candidate for translational application in COPD. Furthermore, RIPK1 inhibitors, including eclitasertib [184], SIR2446M [185], and GFH312 [186] have shown potent target engagement and favorable tolerability in early-phase trials, supporting the feasibility of pharmacologically modulating necroptosis and inflammation through selective kinase inhibition.

## 10. Conclusions

Disruption of apoptosis, necroptosis, pyroptosis, ferroptosis, cuproptosis, and parthanatos is well established as a driver of COPD progression. In contrast, autophagy exhibits a context-dependent dual role: protective in early stages by clearing damaged organelles and alleviating stress, yet potentially detrimental in advanced disease when dysregulated or impaired [187]. This Janus-faced nature of autophagy highlights the importance of considering timing, extent, and microenvironmental context when designing therapeutic strategies that target RCD in COPD.

While distinct molecular mechanisms independently regulate various kinds of RCD, they also have potential interconnections. Multiple RCD pathways can be initiated by common external stimuli, such as ROS, as seen in ferroptosis and parthanatos [18]. Oxeiptosis is another ROS-initiated RCD, though its role in COPD remains unclear. DAMPs and PAMPs that activate pyroptosis can be released by cells undergoing necroptosis [121]. Necroptosis itself can be triggered by apoptosis inhibitors. Additionally, key components regulating different RCDs may overlap. For instance, RIPK1, a central mediator of necroptosis, and AIF, a key player in parthanatos, can also mediate apoptosis [52,188]. Notably, pyroptosis and ferroptosis are closely linked. Inactivation of GPX4 during ferroptosis causes lipid peroxidation that activates caspase 11 and gasdermin D cleavage, thereby inducing pyroptosis. Conversely, activation of the NLRP3 inflammasome during pyroptosis can promote iron accumulation and lipid peroxidation, potentially leading to ferroptosis [189]. Thus, the independent yet intertwined relationships between various RCDs present both opportunities and challenges for targeting RCDs in COPD treatment.

The temporal relationships among different RCD pathways in COPD remain unclear, representing a major conceptual gap. Most studies offer static observations within isolated disease stages, making it difficult to define their sequence or dominance. However, it can be speculated that apoptosis and autophagy likely predominate early in response to cigarette smoke, supporting repair and clearance, while necroptosis, pyroptosis, and ferroptosis become more prominent as oxidative stress and inflammation progress. These pathways may act concurrently rather than sequentially. Time-resolved preclinical studies are needed to clarify the dynamics of these RCD pathways. Insights from such studies may help define diagnostic and therapeutic windows, thereby facilitating the development of stage-specific biomarkers and targeted treatment strategies.

This review summarizes the roles of diverse RCD pathways in the pathogenesis of COPD and highlights the possible therapeutic modulation of these processes. The development of novel COPD treatments may be facilitated by the modification of key components within RCD pathways. However, whether other emerging RCD pathways, like oxeiptosis and alkaliptosis, also play roles in COPD pathogenesis remains an area for future research.

## Figures and Tables

**Table 1:** Agents targeting COPD via regulating apoptosis pathway.

Compound	RCD Types	Target	COPD Model	Functions	Ref.
Anthrahydroquinone-2,6-disulfonate (AH_2_QDS)	Apoptosis	Nrf2, HO-1, NQO1↑	SD rats	Attenuates CS-induced lung inflammation	[126]
Asiaticoside (AS)	Apoptosis	PGC-1α, Nrf2↑	BEAS-2B cells	Reduces CSE-induced TNF-α, IL-6 and EMT	[122]
Formononetin (FMN)	Apoptosis	AhR, CYP1A1, AKT, mTOR↓	BEAS-2B cells	Reduces CSE-induced TNF-α, IL-1β	[124]
Gaylussacin	Apoptosis	SIRT1↑, MMP-12↓	Mice	Attenuates Pb/Cd-induced emphysema and lung inflammation	[127]
	Apoptosis	SIRT1↑, MMP-12↓	MH-S cells	Reduces Pb/Cd-induced TNF-α, IL-1β	[127]
Korean Red Ginseng (KRG)	Apoptosis	Bax, Caspase 3↓; Bcl-2↑	NCI-H292 cells	Reduces CSC-induced TNF-α, IL-1β	[123]
	Apoptosis	Bax, Caspase 3↓; Bcl-2↑	Mice	Attenuates CS-induced emphysema and lung inflammation	[123]
N-acetyl-L-cysteine (NAC)	Apoptosis	p53, Caspase 3↓; Bcl-2, HO-1, GSH↑	RLE-6TN cells	-	[125]
	Apoptosis	p53, Caspase 3↓; Bcl-2, HO-1, GSH↑	Wistar rats	Attenuates CS-induced emphysema and lung inflammation	[125]
Progesterone (P4)	Apoptosis	SIRT1, PGC-1α, Nrf1↑	Mice	Attenuates CS-induced emphysema and lung inflammation	[128]

BEAS-2B: human bronchial epithelial cell; MH-S: murine alveolar macrophage cell; NCI-H292: national cancer institute-H292 cell; RLE-6TN: rat lung epithelial-6 tumor necrosis factor-responsive cell; CS: cigarette smoke; CSE: cigarette smoke extract; IL-6: interleukin 6; IL-1β: interleukin 1β; TNF: tumor necrosis factor. “↓”: down-regulation; “↑”: up-regulation. “-“: none.

**Table 2:** Agents targeting COPD via regulating necroptosis pathway.

Compound	RCD Types	Target	COPD Model	Functions	Ref.
GSK’547	Necroptosis	RIPK1 kinase inhibitor	Mice	Attenuates CS-induced parenchymal inflammation, airway remodeling and emphysema	[52]
Theaflavin-3,3′-digallate (TF-3)	Necroptosis	p-RIPK3, p-MLKL↓	Mice	Attenuates CS-induced emphysema and lung inflammation	[131]
GSK’872	Necroptosis	RIPK3 kinase inhibitor	Mice	Attenuates CS-induced emphysema and lung inflammation	[56]
	Necroptosis	RIPK3 kinase inhibitor	MLE-12 cells	Reduces CSE-induced TNF-α, IL-6 and cell death	[56]
	Necroptosis	RIPK3 kinase inhibitor	BMDMs	Reduces CSE-induced CXCL1, CXCL2, IL-6 and cell death	[51]
Necrostatin-1 (NEC-1)	Necroptosis	RIPK1 kinase inhibitor	BMDMs	Reduces CSE-induced CXCL1, CXCL2, IL-6 and cell death	[51]
	Necroptosis	RIPK1 kinase inhibitor	Mice	Suppresses the CS-induced neutrophilic airway inflammation	[121]
Thymoquinone (Tq)	Necroptosis	p-MLKL, RIP-1, RIP-3↓	BEAS-2B cells	Reduces cell death	[132]

BEAS-2B: human bronchial epithelial cell; CS: cigarette smoke; CSE: cigarette smoke extract; IL-6: interleukin 6; CXCL-1: C-X-C motif chemokine ligand 1; CXCL-2: C-X-C motif chemokine ligand 2; TNF: tumor necrosis factor. “↓”: down-regulation.

**Table 3:** Agents targeting COPD via regulating ferroptosis pathway.

Compound	RCD Types	Target	COPD Model	Functions	Ref.
Ginkgo biloba extract (GBE)	Ferroptosis	GSH, GPX4, FTH1↑; ACSL4↓	Mice	Alleviates PM2.5-induced emphysema and airway inflammation	[141]
Acacetin	Ferroptosis	GSH, GPX4, SLC7A11↑; Fe^2+^↓	16HBE cells	Decreases cell death	[143]
Ginsenoside Rg1	Ferroptosis	GSH, GPX4↑; Fe^2+^↓	BEAS-2B cells	Reduces CSE-induced IL-6, TNF-α and IL-1β	[140]
Ethyl acetate fraction of Thesium chinense Turcz (TCEA)	Ferroptosis	GPX4, SLC7A11↑; ACSL4, ALOX15↓	Mice	Attenuates CS-induced emphysema and lung inflammation	[133]
Fluorofenidone (AKF)	Ferroptosis	GSH, GPX4, SLC7A11↑; MDA, Fe^2+^↓	BEAS-2B cells	Reduces CSE-induced IL-6, TNF-α and IL-1β	[134]
	Ferroptosis	GSH, GPX4, SLC7A11↑; MDA, Fe^2+^↓	Mice	Attenuates CS-induced emphysema, lung fibrosis and lung inflammation	[134]
Sea buckthorn extract (SBE)	Ferroptosis	SLC7A11, GSH, GPX4↑; MDA, ACSL4↓	Mice	Alleviates CS-induced emphysema and airway inflammation	[142]
Sodium pyruvate (NaPyr)	Ferroptosis	GSH, GPX4, NRF2↑; COX2↓	A549 and BEAS-2B cells	Reduces CSE-induced TNF-α and IL-8	[145]
Ferrostatin-1 (Fer-1)	Ferroptosis	NRF2, GPX4↑; COX2↓	A549 and BEAS-2B cells	Reduces CSE-induced TNF-α and IL-8	[145]
	Ferroptosis	Fe^2+^, ALOX15↓	Mice	Attenuates LPS/CS-induced airway inflammation and MUC5AC	[36]
	Ferroptosis	GPX4↑; COX2↓	BEAS-2B cells	alleviates PM2.5-induced IL-6, IL-8, TNF-α	[135]
	Ferroptosis	GPX4, SLC7A11↑	BEAS-2B cells	Reduces CSE-induced TNF-α, IL-6 and cell death	[136]
	Ferroptosis	GPX4, SLC7A11↑	Bronchoalveolar epithelial cells (BAECs)	Reduces CSE-induced TNF-α, IL-6 and cell death	[137]
	Ferroptosis	GPX4↑	16HBE cells	Decreases cell death	[137]
	Ferroptosis	GPX4↑	BEAS-2B cells	Decreases cell death	[138]
Scutellarein (STR)	Ferroptosis	GPX4, NRF2↑; ALOX15↓	Mice	Attenuates LPS/CS-induced airway inflammation and MUC5AC	[36]
Sodium hydrosulfide	Ferroptosis	NRF2, GPX4, NCOA4, PPAR-γ↑; COX2↓	Mice	Alleviates PM2.5-induced emphysema and airway inflammation	[135]
	Ferroptosis	NRF2, GPX4, NCOA4, PPAR-γ↑; COX2↓	BEAS-2B cells	Alleviates PM2.5-induced IL-6, IL-8, TNF-α	[135]
Deferoxamine (DFO)	Ferroptosis	GPX4↑, COX2↓	BEAS-2B cells	Alleviates PM2.5-induced IL-6, IL-8, TNF-α	[135]
	Ferroptosis	GPX4, SLC7A11↑	BEAS-2B cells	Reduces CSE-induced TNF-α, IL-6 and cell death	[136]
	Ferroptosis	GPX4↑	16HBE cells	Decreases cell death	[62]
Dihydroquercetin (DHQ)	Ferroptosis	GPX4, SLC7A11↑	Mice	Alleviates CS-induced emphysema	[148,149]
Curcumin (CUR)	Ferroptosis	GPX4, SLC7A11↑	BEAS-2B cells	Reduces CSE-induced TNF-α, IL-6 and cell death	[136]

16HBE: human airway epithelial cell; A549: alveolar epithelial cell; BEAS-2B: human bronchial epithelial cell; CS: cigarette smoke; CSE: cigarette smoke extract; LPS: lipopolysaccharide; PM: particulate matter; IL-6: interleukin 6; IL-8: interleukin 8; IL-1β: interleukin 1β; TNF: tumor necrosis factor. “↓”: down-regulation; “↑”: up-regulation.

**Table 4:** Agents targeting COPD via regulating autophagy pathway.

Compound	RCD Types	Target	COPD Model	Functions	Ref.
Bufei Yishen Formula (BYF)	Autophagy	LC3BII↑, p62↓	16HBE cells	Reduces CSE-induced IL-1β, IL-6, TNF-α, MMP-2, MMP-9	[148]
	Autophagy	LC3BII↑	BEAS-2B cells	Reduces CSE-induced IL-1β, IL-6, TNF-α; maintains epithelial barrier integrity	[149]
Dihydromyricetin (DHM)	Autophagy	Beclin1, LC3BII↑, p62↓	Airway organoids	Reduces mucus hypersecretion and repairs ciliary function	[150]
Diindolylmethane (DIM)	Autophagy	LC3BII↓, p62↑	HEL299 cells	Reduces CdCl_2_-induced oxidative stress	[153]
Quercetin	Autophagy	p62↑; ROS, LC3BII, MDA↓	SD rats	Alleviates CS-induced collagen deposition and airway inflammation; improves lung function	[151]
Vardenafil	Autophagy	Beclin1, LC3BII↑, p62↓	Mice	Alleviates CS-induced emphysema and airway inflammation	[152]
Hydrogen sulfide (H_2_S)	Mitophagy	PINK1, Parkin↑, ROS, MDA↓	Mice	Alleviates CS-induced emphysema and airway inflammation; improves lung function	[88]
Puerarin	Mitophagy	PINK1, Parkin, DRP1, FUNDC1↓	16HBE cells	Reduces CSE-induced cell death and restores mitochondrial function	[154]

16HBE: human airway epithelial cell; BEAS-2B: human bronchial epithelial cell; HEL299: human embryonic lung fibroblast 299 Cell; CS: cigarette smoke; CSE: cigarette smoke extract; MMP-2: matrix metallopeptidase 2; MMP-9: matrix metallopeptidase 9; IL-6: interleukin 6; IL-1β: interleukin 1β. “↓”: down-regulation; “↑”: up-regulation.

**Table 5:** Agents targeting COPD via regulating pyroptosis pathway.

Compound	RCD Types	Target	COPD Model	Functions	Ref.
Tianlong kechuanling (TL)	Pyroptosis	NLRP3 inflammasome activation↓; GSDMD-N↓	Mice	Alleviates LPS + CS + Hx-induced lung function decline, pulmonary hypertension and airway inflammation	[166]
Resveratrol	Pyroptosis	NLRP3 inflammasome activation↓; GSDMD-N↓; NRF2/HO-1↑	BEAS-2B, 16HBE and A549 cells	Reduces CSE-induced IL-18, IL-1β and cell death	[155]
Dihydromyricetin	Pyroptosis	NLRP3 inflammasome activation↓; GSDMD-N↓; SLC7A11, GPX4↑	Mice	Alleviates LPS + CS-induced lung function decline and airway inflammation	[156]
Disulfiram (DSF)	Pyroptosis	GSDMD-N↓	Mice	Alleviates O3-induced emphysema and airway inflammation; maintains epithelial barrier integrity	[167]
Astragaloside IV	Pyroptosis	NLRP3 inflammasome activation↓; GSDMD-N↓	BEAS-2B cells	Reduces cell death	[165]
	Pyroptosis	NLRP3 inflammasome activation↓; GSDMD-N↓	Mice	Alleviates LPS + CS-induced emphysema and airway inflammation	[165]
Propofol	Pyroptosis	NLRP3 inflammasome activation↓; GSDMD-N↓; NRF2↑	16HBE cells	Ameliorates CSE-induced IL-6, TNF-α, IL-1β and reduces cell death	[168]
Schisandrin A (SchA)	Pyroptosis	NLRP3 inflammasome activation↓; GSDMD-N↓; NRF2/HO-1↑	Mice	Alleviates CS-induced emphysema and airway inflammation; improves lung function	[157]
Tanshinone (TS)	Pyroptosis	-	Mice	Alleviates LPS + CS + H1N1-induced lung function decline and airway inflammation	[158]
	Pyroptosis	NLRP3 inflammasome activation↓; NF-κB signaling activation↓	BEAS-2B and Raw264.7 cells	Alleviates LPS + CSE-induced IL-6, IL-8, TNF-α and IL-1β	[158]
grape seed proanthocyanidin extract (GSPE)	Pyroptosis	-	Mice	Ameliorates lung inflammation and emphysema induced by intraperitoneal injection of CSE.	[159]
	Pyroptosis	NLRP3 inflammasome activation↓	RAW 264.7 cells	-	[159]
Daphnetin (Daph)	Pyroptosis	NLRP3 inflammasome activation↓; GSDMD-N↓	Mice	Alleviates PM2.5/PM2.5 + CS-induced airway inflammation and hypersecretion	[160]
	Pyroptosis	NLRP3 inflammasome activation↓; GSDMD-N↓	BEAS-2B	Alleviates PM2.5/PM2.5 + CSE-induced IL-1β and cell death	[160]
MCC950 (Also known as cRId3)	Pyroptosis	NLRP3 inflammasome activation↓; GSDMD-N↓	Mice	Alleviates PM2.5/PM2.5 + CS-induced airway inflammation	[160]
	Pyroptosis	The NLRP3 inhibitor	16HBE cells	Reduces CSE-induced IL-1β and TLR4	[169]
	Pyroptosis	The NLRP3 inhibitor	Mice	Alleviates CS-induced airway inflammation	[170]
Osthole	Pyroptosis	NLRP3 inflammasome activation↓	16HBE cells	Reduces CSE-induced IL-6, TNF-α and IL-1β	[161]
The flower buds of *Tussilago farfara* L. (FTF)	Pyroptosis	NLRP3 inflammasome activation↓	Mice	Alleviates CS-induced airway inflammation	[162]
Hydrogen sulfide	Pyroptosis	NLRP3 inflammasome activation↓; GSDMD-N↓	SD rats	Attenuates CS-induced lung inflammation	[169]
	Pyroptosis	NLRP3 inflammasome activation↓; GSDMD-N↓	16HBE cells	Reduces CSE-induced IL-1β	[169]
	Pyroptosis	NLRP3 inflammasome activation↓	Mice	Attenuates CS-induced airway inflammation and emphysema	[171]
	Pyroptosis	NLRP3 inflammasome activation↓	A549 cells	Alleviates PM2.5-induced IL-1β and cell death	[171]
VX-765	Pyroptosis	Specific Caspase-1 inhibitor	16HBE cells	Alleviates PM2.5-induced IL-1β, IL-6, IL-8, CXCL-1 and CXCL-2	[91]
	Pyroptosis	Specific Caspase-1 inhibitor	16HBE cells	Alleviates CSE-induced IL-1β, IL-18 and cell death	[105]
Magnesium isoglycyrrhizinate (MgIG)	Pyroptosis	NLRP3 inflammasome activation↓	Wistar rats	Alleviates LPS + CS-induced lung function decline, airway inflammation, airway remodeling and emphysema	[172]
Isoforskolin (ISOF)	Pyroptosis	NLRP3 inflammasome activation↓	Mice	Alleviates CS + H1N1-induced lung function decline and airway inflammation	[164]
(−)-Epicatechin (EC)	Pyroptosis	NLRP3 inflammasome activation↓; GSDMD-N↓	Rats	Alleviates CS-induced lung inflammation and emphysema	[163]
	Pyroptosis	NLRP3 inflammasome activation↓; GSDMD-N↓	BEAS-2B cells	Reduces CSE-induced IL-18, IL-1β and cell death	[163]

16HBE: human airway epithelial cell; A549: alveolar epithelial cell; BEAS-2B: human bronchial epithelial cell; RAW 264.7: mouse mononuclear macrophage; CS: cigarette smoke; CSE: cigarette smoke extract; LPS: lipopolysaccharide; PM: particulate matter; IL-6: interleukin 6; IL-8: interleukin 8; IL-1β: interleukin 1β; IL-18: interleukin 18; CXCL-1: C-X-C motif chemokine ligand 1; CXCL-2: C-X-C motif chemokine ligand 2; TNF: tumor necrosis factor. “↓”: down-regulation; “↑”: up-regulation. “-“: none.

## Data Availability

No new data were created or analyzed in this study.

## Abbreviations

Regulated cell death (RCD), chronic obstructive pulmonary disease (COPD), accidental cell death (ACD), mitochondrial outer membrane permeabilisation (MOMP), cytochrome c (Cyto-C), TNF receptor superfamily member 1A(TNFRSF1A), death-inducing signal complex (DISC), Unc-5 Netrin Receptor B (UNC5B), DCC Netrin 1 Receptor (DCC), death-associated protein kinase 1 (DAPK1), cigarette smoke extract (CSE), epithelial–mesenchymal transition (EMT), unfolded protein response (UPR), human airway smooth muscle cells (HASMCs), damage-associated molecular patterns (DAMPs), TNF receptor-associated death domain protein (TRADD), phosphorylated RIPK3 (p-RIPK3), phosphorylated MLKL (p-MLKL), mitochondrial DNA (mtDNA), Ferric iron (Fe^3+^), transferrin receptors (TFRs), ferrous iron (Fe^2+^), glutathione (GSH), phospholipid hydroperoxides (PL-OOH), glutathione peroxidase 4 (GPX4), oxygen species (ROS), polyunsaturated fatty acids (PUFAs), Autophagic cell death (ACD), Nomenclature Committee on Cell Death (NCCD), autophagy-related (ATG), UNC-51-like kinase 1 (ULK1), phosphatidylinositol 3-phosphate (PI3P), light chain 3 (LC3), phosphatidylethanolamine (PE), polystyrene microplastics (PS-MPs), autophagy-dependent ferroptosis (ADF), gasdermin (GSDM), pathogen-associated molecular patterns (PAMPs), leucine-rich repeat-containing proteins (NLRs), N-terminal fragment (GSDM-N), lipopolysaccharide (LPS), apoptosis-inducing factor (AIF), Endonuclease G (EndoG), cigarette smoke condensate(CSC), porcine pancreatic elastase (PPE), bone marrow-derived macrophages (BMDMs), acute respiratory distress syndrome (ARDS), dimethyl fumarate (DMF), metabolite monomethyl fumarate (MMF).
